# Safety and Effectiveness of the Prolonged Treatment of Children with a Ketogenic Diet

**DOI:** 10.3390/nu12020306

**Published:** 2020-01-24

**Authors:** Jana Ruiz Herrero, Elvira Cañedo Villarroya, Juan José García Peñas, Beatriz García Alcolea, Begoña Gómez Fernández, Laura Andrea Puerta Macfarland, Consuelo Pedrón Giner

**Affiliations:** 1 Department of Pediatric Gastroenterology, Pediatric Service, San Rafael Hospital, 28016 Madrid, Spain; 2 Department of Gastroenterology and Nutrition, Niño Jesús Pediatric Hospital, 28009 Madrid, Spain; 3 Department of Neurology, Niño Jesús Pediatric Hospital, 28009 Madrid, Spain

**Keywords:** ketogenic diet, drug-resistant epilepsy, GLUT-1, growth

## Abstract

Background: The ketogenic diet (KD) is an effective treatment against drug-resistant epilepsy in children. The KD is a diet rich in fats that produces anticonvulsant and neuroprotective effects that reduces seizures and improves the cognitive state. Nevertheless, it can produce side effects that sometimes can be serious. Further, the effect on growth is quite controversial when used for an extended period of time. The aim of this paper was to assess the effectiveness, side effects, and repercussions in the development of children who have been treated with a KD for more than 2 years. Methods: Observational descriptive study of 26 pediatric patients on a KD, with data collection at baseline, at 3, 6, and 12 months, and then once a year. Number of seizures, type of seizures, anti-seizure drugs, anthropometry, side effects, and alterations in laboratory assessment were monitored. Results: In every assessment, about 60%–75% of the patients experienced a reduction in number of seizures of over 90%, and at least 50% experienced side effects, of which digestive issues, alteration in the lipid metabolism, and hypercalciuria were the most common. The KD significantly affected height after 2 years of treatment. Conclusions: The KD is an effective treatment for drug-resistant epilepsy. Its side effects, although common, are very mild; therefore, this constitutes a very safe treatment for children of all ages. More studies are needed to identify and prevent potential causes of growth retardation in children on the KD.

## 1. Introduction

A ketogenic diet (KD) is characterized by the high percentage of calories obtained from fat and a limited percentage obtained from carbohydrates [1]. There are different types of diets according to the different amounts of macronutrients [1,2]. In the classic KD, 87%–90% of daily caloric intake comes from fat sources [2]. The provision of carbohydrates and proteins may vary within ratios of 3:1 to 4:1 (3–4 g of fat to 1 g of carbohydrates plus protein). In the KD with medium-chain triglyceride (MCT, KD-MCT) approximately 71% of caloric intake comes from fat sources, of that, 11% is from natural foods and 60% is from MCT [3]. There is another KD-MCT in which natural food fat percent is higher (41%) and instead, only 30% of fat comes from MCT. The modified Atkins diet (MAD) allows an unrestricted amount of protein and fat but does restrict the carbohydrate intake to 10 g/day [4,5]. Lastly, the low glycemic index diet (LGID) is rich in fat but allows for a higher amount of carbohydrates, permitting only those foods whose glycemic index is lower than 50 [6].

The KD is an effective treatment against drug-resistant epilepsy [7], defined by the International League Against Epilepsy (ILAE) as “uncontrolled epilepsy in which two or more drugs have been used for treatment in the correct dosage”. The KD works through different mechanisms, complementary and not fully known. These include ketosis; reduced glucose; elevated fatty acid levels; enhanced bioenergetic reserves; and others with anticonvulsant, anti-epileptogenic, and neuroprotective properties [8]. The KD must be considered precociously as treatment in specific epileptic syndromes and in some etiologies where it is known to be very effective [9,10], including Dravet syndrome [11,12], infantile spasms and West syndrome [13,14], Doose syndrome [15,16], tuberous sclerosis complex [17], and FIRES syndrome (febrile infection related epilepsy syndrome) [18,19]. Moreover, it is the treatment of choice in the deficiency of glucose type 1 transporter (GLUT-1) [20] and in pyruvate dehydrogenase deficiency (PDH) [21].

Side effects may occur at the beginning of the diet as well as months after the introduction of the diet. Short-term side effects are dehydration, electrolyte abnormalities, hypoglycemia, lethargy, hyperuricemia, acidosis, abdominal pain, nausea/vomiting, diarrhea, constipation, hepatitis, or acute pancreatitis [22,23]. Long-term side effects could be hypoproteinemia, hypocalcemia, hypercalciuria, urolithiasis, lipid profile changes, transaminase increase, or cardiomiopathy [22,23]. There is little information about possible long-term side effects in children on the KD for longer than 2 years: increased risk of bone fractures, kidney stones, and growth delay are the most referred [24]. In most cases, the symptomatology experienced is often mild and self-limited, and only in a small percentage of patients are dietary modifications or pharmacological treatment needed, and in rare and severe cases the patient must be weaned off the KD. KD is not a balanced diet, so the intake of vitamins and minerals can be compromised. Nutrients deficiency has been described (vitamin D, selenium, magnesium, iron, and carnitine) [22,25,26]. The effect on growth is quite controversial. Some studies show no change in growth patterns [27,28], whereas studies where a longer follow-up was done for a longer period of growth did seem to be affected [29,30], especially in younger children [31]. Two very recent papers suggest a protective effect of citrate supplementation [32,33].

The main goal of this study was to assess the effectiveness of long-term KD, as well as the side effects, especially those with regard to growth development.

## 2. Materials and Methods

Data of all pediatric patients (<18 years old) who had been prescribed a KD in a Spanish tertiary care level hospital in Madrid between January 2000 and December 2018 were collected. It is a retrospective and prospective (since May 2015) observational descriptive study. It was approved by the Committee for Ethics in Clinical Research of the University Children’s Hospital Niño Jesús (R-0002/15) as part of a more complete investigation to determine the changes in gene expression and metabolism induced by a KD. All patients or their relatives were fully informed of the aims and scope of the research and signed an informed consent. All data were collected anonymously. The inclusion criteria were being under 18 years old and having been treated for at least 2 years with a KD. A total of 26 participants were recruited for this study. The age at the beginning of the KD ranged from 3 months to 12.5 years. Five patients were infants ≤1 year old at the onset of a KD, nine were 1–5 years old, nine were 5–10 years old, and three were older than 10 years. Full medical histories were reviewed in order to collect epidemiological data, information about the disease they suffered, type and amount of seizures, and treatments they had received prior to the diet´s implementation, including drug use. Antiepileptic drugs (AEDs) were prescribed by the neurologist on the basis of the patient’s weight, and these were adjusted according to clinical changes and blood levels, if any.

To estimate the resting energy expenditure, an indirect calorimetry was performed for only four patients. The recommendations of the World Health Organization on the basis of age and weight were used for the remainder of patients [34]. Protein intake was calculated according to daily protein recommendations (generally 1 g per kilo per day in children over 1 year and 1.5 in children under 1 year) [35,36]. Needs of liquids were calculated according to Holliday–Segar method [37]. Infants younger than 6 months were on a liquid diet based on Ketocal^®^. Older infants and children were on a diet based on natural foods supplemented with a fat emulsion consisting of MCT (Liquigen^®^) or with powders or liquids rich in lipids and low in carbohydrates, based on whey protein supplemented with amino acids and micronutrients (Ketocal ^®^). Only when an insufficient intake or a nutritional deficiency was found were the children started on a carbohydrate-free multivitamin, or a calcium, vitamin D, or other supplement. The dosage was to reach Recommended Dietary Allowance (DRA) or greater if necessary.

All patients underwent a thorough examination prior to the KD and then after the 1st, 3rd, 6th, and 12th month and then yearly after being weaned off the diet or being discharged. The effectiveness of the diet was measured by a reduction in AEDs and the number of seizures. The effect of the KD on the seizures was assessed on the basis of the percentage of incidence: 100% (lack of seizures), 90%–100% improvement, 50%–90%, <50%, 0% (no improvement), or increase of the amount of seizures [38].

In each medical examination, possible side effects were accounted for and were divided into early (if they appeared within the first month) or late (if they appeared after the first month). Anthropometric measures were likewise analyzed (weight, height, and body mass index (BMI)) in order to identify growth abnormalities. It was considered normal if the z-score of the BMI was between −2 and +1 in children older than 5 years. Mild malnutrition if z was <−2 and ≥−3 and severe malnutrition if z < −3. Overnutrition was divided into three categories (overweight, >+1 and ≤+2; obesity, z > +2; and ≤ +3 and severe obesity, z > +3). In children younger than 5 years, normalcy was established between −2 and +2, malnutrition was considered to have the same parameters stated above, and overnutrition was as follows: overweight z > +2 and ≤ +3, and obesity z > +3. Parameters were adjusted by sex and age according to the growth charts of the World Health Organization [39,40,41]. Height development was considered normal when the z score was between −2 and +2, mild alteration if z was <−2 and ≥−3, and severe alteration if z was <−3. Laboratory assessment, in order to identify side effects and evaluate nutritional status, included complete blood count; biochemical profile; lipid profile; gasometry; ions; urinary sediment; calcium/creatinine relation; protein/creatinine and citrate/creatinine; prealbumin and retinol binding protein (RBP); vitamins A, E, D, and B12; folic acid; zinc; selenium; parathormone (PTH); and carnitine. Then, we specified, between parentheses, the normal ranges and degrees of deficiency of each of the parameters evaluated. Prealbumin (>15 mg/dL, mild decrease 10–15, moderate 5–10, and severe <5 mg/dL); RBP (>2.5 mg/dL, mild decrease 2–2.5 mg/dL, moderate 1.5–2 mg/dL, and severe <1.5 mg/dL); vitamin A (0.2–0.5 mg/L); vitamin E (0.8–10 mg/g of lipids); vitamin D (20–60 ng/mL); ferritin (7–140 ng/mL); vitamin B12 (>250 pg/mL); folic acid (>3.9 ng/mL); cholesterol (<200 mg/dL); triglycerides (<115 mg/dL); aspartate aminotransferase (AST) and alanine aminotransferase (ALT) (<50 IU/L); gamma glutamyl transpeptidase (GGT) (<35 IU/L); uric acid (<6 mg/dl); calciuria (calcium/creatinine in urine < 0.2 mg/mg in children older than 2 years and <0.5 in under 2 years); pH in gasometry (>7.3); bicarbonate (>20 mMol/L); zinc (>70 mcg/dL); selenium (>70 mcg/L); carnitine (>33 mcM/L); and magnesium (>1.5 mg/dL).

## 3. Statistical Analysis

Variables were registered in an Excel program table. All statistical analyses were carried out with R software version 3.5.2. Figures were generated with R and Excel software. Prior to the main statistical analyses, the normality assumption of studied variables was confirmed using the Shapiro–Wilk test. Paired sample Student’s *t*-test was used to assess significant differences in the z-scores for weight, height, and BMI at baseline and at 24 months of KD treatment for the complete cohort. The effect of the introduction of the KD in patients younger and older than 2 years was also assessed, creating two age groups. The unpaired Wilcoxon signed-rank test was used to compare the z-score values for weight, height, and BMI for these to age groups after 2 years of treatment with a KD. A two-tailed test was performed, and *p* < 0.05 was considered significant.

## 4. Results

The study consisted of 26 patients (9 females and 17 males) on a KD. In Table 1, the characteristics of the sample are specified. Of the 26 cases, 25 suffered from drug-resistant epilepsy and the remaining patient’s initial diagnosis was GLUT-1 deficiency, which was not genetically confirmed. This child suffered from movement disorder, dystonia, and ataxia. Two years after the introduction of the KD, a *NALCN* (sodium leak channel, non-selective) gene mutation was perceived compatible with congenital contractures of the limbs and face with hypotonia and developmental delay syndrome (CLIFAHDD syndrome). The causes for epilepsy are shown in Table 2. There was one case of Doose syndrome, one of Otahara syndrome, and one of Lennox–Gastaut syndrome among the four patients in which no cause was identified. The average number of AEDs used before the diet was 3.72 (range of 0–11), and 15 out of the 25 patients suffered side effects due to medication. None of the patients underwent surgery, and one improved as a result of having a vagus nerve stimulator. Other treatments that have not been effective were previously used: KD already withdrawn (1 patient), corticoids (1), gamma globulins (1), cofactors (1), adrenocorticotropic hormone (ACTH) (2), and pyridoxine (8). 

Two patients were in status epilepticus before starting on the KD, and three patients could not specify the quantity of seizures per day, but were numerous in quantity, whereas one patient suffered none. Out of the other 20 patients, 3 did not have daily seizures (ranging from 1 per month to 4 per week) and 17 had daily seizures (ranging from 1 to 60 seizures per day). The average amount of AEDs administered prior to the KD was 2.8 (median 3; maximum 5). Electroencephalogram recording was performed for 21 patients before the KD, and in all cases the results were abnormal. 

Four patients were infants at the onset of the diet. In all of these cases, a CKD with a 3:1 ratio was administered, and in 1 case the administration route was through nasogastric tube. All patients who received a 4:1 ratio diet were older than 3 years, and in three of these cases the administration route was not oral (2 g-tubes and 1 nasogastric tube). These diets were introduced slowly and progressively without fasting and without liquid restriction. One exception was one patient who was in status epilepticus whose introduction of the KD was done quickly and who had a calorie restriction of 25% of the daily recommended intake. In six of the cases, they were out-patients and all were on a MAD. Two patients started their diet in another hospital. The rest of the sample started the diet in the Hospital Niño Jesús. Average ketosis was ≥2.4 mM/L and it was achieved in a median of 4 days (1–30 days).

During the follow-up, one child needed the administration of the diet through a g-tube (1.5 months after starting the diet) and there were changes in the type of diet and of ratio in 11 cases: one patient went from having a 3:1 ratio to a 2:1 ratio on the fourth day; two patients went from a 4:1 ratio to a 3:1 ratio on the 7th and on the 43rd day due to side effects; four patients went from having a CKD to a MAD (one due to side effects, three due to the long duration of the diet); and four patients went from having a MAD to a LGID also due to the long duration of the diet. The average of β-hydroxybutyrate blood levels during follow-up were 3 months, 2.23 mM/L (0–5.9); 6 months, 2.7 (0.2–5.1); 12 months, 3.14 (0.1–5.9); 2 years, 2.9 (0.3–5.9); 3 years, 2.8 (0.3–4.6); 4 years, 2.41 (0.4–6.7); 5 years, 2.47 (1.2–3.7); 6 years, 1.23 (0.9–1.4). 

The median length on a KD was 3.91 years. Most often the reason to suspend the diet was the long duration (8/10). In one case, the diet was suspended due to dietary treatment of eosinophilic esophagitis, the seizures in the beginning got worse but the child later returned to a normal state. In another case, the diet was removed because GLUT-1 deficiency was suspected initially but later a Christianson syndrome was confirmed with an SLC9AC gene mutation. 

### 4.1. Outcome of the KD

**Figure 1:**
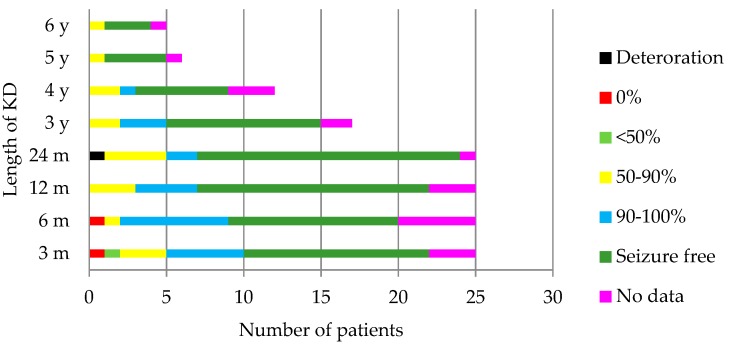
Evolution of the seizures throughout the follow-up. In one patient, the seizures were hard to account for because they happened during sleep. KD: ketogenic diet, m: months, y: years.

Figure 1 shows the results of the amount of seizures in the 25 children who had epilepsy. The following patients had a 100% reduction of the seizures: at 3 months, 12 out of 25 children (48%); at 6 months, 11/25 (44%); at 12 months, 15/25 (60%); at 24 months, 17/25 (68%); at 3 years, 10/17 (58.8%); at 4 years 7/12 (58.3%); at 5 years 4/6 (66.6%); at 6 years 3/5 (60%). The reduction of the seizures was higher than 90% in 68% of the children 3 months after the diet, 72% after 6 months, 76% after 12 and 24 months and 3 years, 66.6% after 4 and 5 years, and 60% after 6 years. Table 3 shows the effectiveness of the diet according to the etiology of epilepsy. The patient with a movement disorder had a noticeable improvement of her symptomatology. A subjective cognitive or behavioral improvement was observed in 10 (38.46%), 9 (34.61%), and 8 (30.76%) patients at 6, 12, and 24 months, respectively. The patients who were able to reduce the number of AEDs taken prior to the diet were 3 months, 6/25; 6 months, 5/25; 12 months, 6/25; 2 years, 9/25; 3 years, 2/17; 4 years, 2/11; 5 years, 1/7; 6 years, 0/6. After 2, 3, and 4 years, one patient did not take any AEDs; after 5 years, two children did not take any AEDs. 

Six patients remained on the KD for more than 6 years. The etiology was very different in each case (two GLUT-1 deficiency syndrome, one of them not confirmed by genetic testing, one due to meningoencephalitis, one CLIFAHDD syndrome, one Leigh syndrome, and one possible mitochondrial disease). One patient did not suffer any seizures before the introduction of the diet and had a CKD with a 3:1 ratio. After commencing the diet, the remaining patients achieved a 100% reduction of the seizures except for one patient who had a reduction of 50%–90%. They were all on a MAD, except for the patient with Leigh syndrome who was on a CKD 4:1 ratio. They all still took 1 to 3 AEDs. 

The patient who remained on the diet the longest time was a male with GLUT-1 deficiency syndrome who was on a MAD for more than 9 years. Before being discharged to another hospital due to his age, the patient was still on valproic acid treatment and had not had seizures since the start of the diet. 

### 4.2. Side Effects

Of the 26 patients, 11 had early onset side effects: nausea and vomiting (4), constipation (3), hypoglycemia (3), hypercalciuria (3), fatigue (2), acidosis (1), diarrhea (1), hypocalcemia (1), and hypertriglyceridemia (1). In most cases they were mild, except in four patients, for which a change of diet was prescribed. 

In the long term, some side effects were detected; most were easy to manage and a change of diet was not needed. These are shown in detail in Table 4. The most altered results in blood parameters were cholesterolemia 389 mg/dl at 3 months; triglyceridemia 496 mg/dl at 3 months; AST 223 IU/L, ALT 310 IU/L, and GGT 111 IU/L in a patient at 12 months on a KD; calciuria 1.15 mg/mg at 12 months; proteinuria 1.2 mg/mg at 12 months; hyperuricemia 8.7 mg/dl at 2 years; pH 7.22 at 2 years. After 12 months, hypercalciuria was associated to a nephrocalcinosis in an ultrasound test; this was the patient who had CLIFAHDD syndrome, in which there was a mutation of the sodium channels. 

### 4.3. Nutritional Evolution

#### 4.3.1. Nutritional Status before the KD

It was possible to calculate the BMI in 22 children prior to the KD. Twelve patients were older than 5 years. They all met the criteria for the BMI z-score. Fourteen of them were younger than 5 years at the beginning of the diet, and all had a normal BMI z-score (except for one with severe malnutrition). The cephalic perimeter was measured in 10 children, 4 of them had z-scores of <−1 (ranging from −2.04 to −3.27). Only three patients had a calorimetry measurement taken and testing positive for hypometabolism. In 19/26 patients, we had access to the data of dietary intake survey, and three of these were found to have deficiency in both vitamins and minerals. The average distribution of macronutrients was 15.70% of protein, 39.68% lipids, and 41.88% carbohydrates.

The average levels of prealbumin and RBP was 20.63 (range of 12.6–54.6) and 3.55 (1.69–6.8) mg/dL, respectively, before the beginning of the KD. Six patients were found to be under a normal range. 

The average results of cholesterol were 161 mg/dL, and only five patients had levels >200 mg/dL (maximum of 354 mg/dL). The average count of triglycerides was 65 mg/dl (range of 23–140 mg/dL). 

Of 24 patients for which it was possible to get a blood count, none of them had anemia. Of the 22 children for which the values of ferritin were tested, all were found to be normal, and for those of whom we had data, neither had B12 nor folic acid deficiency (average values were 833 pg/mL and 12.31 ng/mL, respectively).

The average values for liposoluble vitamins were vitamin A at 0.35 mg/L (range of 0.22–0.64), efficient vitamin E at 5.08 mg/g of lipids (range 3.13–7.5), and vitamin D at 25.18 ng/mL (range 4.53–45.6). Six patients had levels below 20 ng/mL, and all of them were supplemented with cholecalciferol according to DRA (400 IU/day for children ≤1 year old and 600 IU/day for children >1 year old). One of these patients suffered a secondary hyperparathyroidism with vitamin D value of 4.53 ng/mL and a PTH of 176 pg/mL. He was treated with cholecalciferol 3000 IU/day for three months and then supplemented according to RDA. Calcium intake was also ensured (30–75 mg/kg/day). No calcium, phosphorus, or magnesium deficiencies were registered (average value of 9.48 mg/dL, 5.08 mg/dL, and 2.20 mg/dL, respectively). Patients who had their zinc, selenium, and carnitine levels determined, averaged 89.62 microg/dl, 66.17 microg/L, and 40.62 microM/L, respectively. One patient developed a zinc deficiency, six registered a selenium deficiency, and one had a carnitine deficiency. In cases where a deficit was observed, the supplements were prescribed according to the following doses: elemental zinc, 5 mg twice a day for three months; selenium 1–2 mcg/kg (maximum 60 mcg) divided into two doses for 3 months; carnitine daily at 50 mg/kg. 

#### 4.3.2. Nutritional Status after the Introduction of the KD

**Figure 2:**
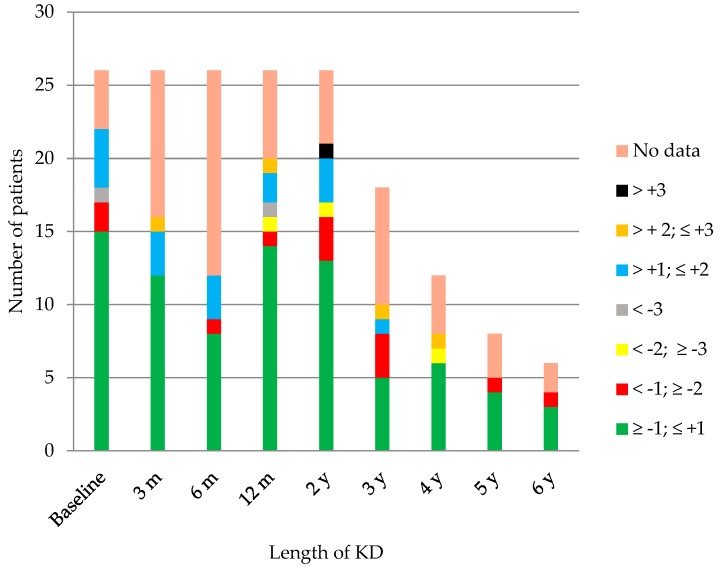
Number of patients in each range of the body mass index (BMI) z-score throughout time. KD: ketogenic diet, m: months, y: years.

**Figure 3:**
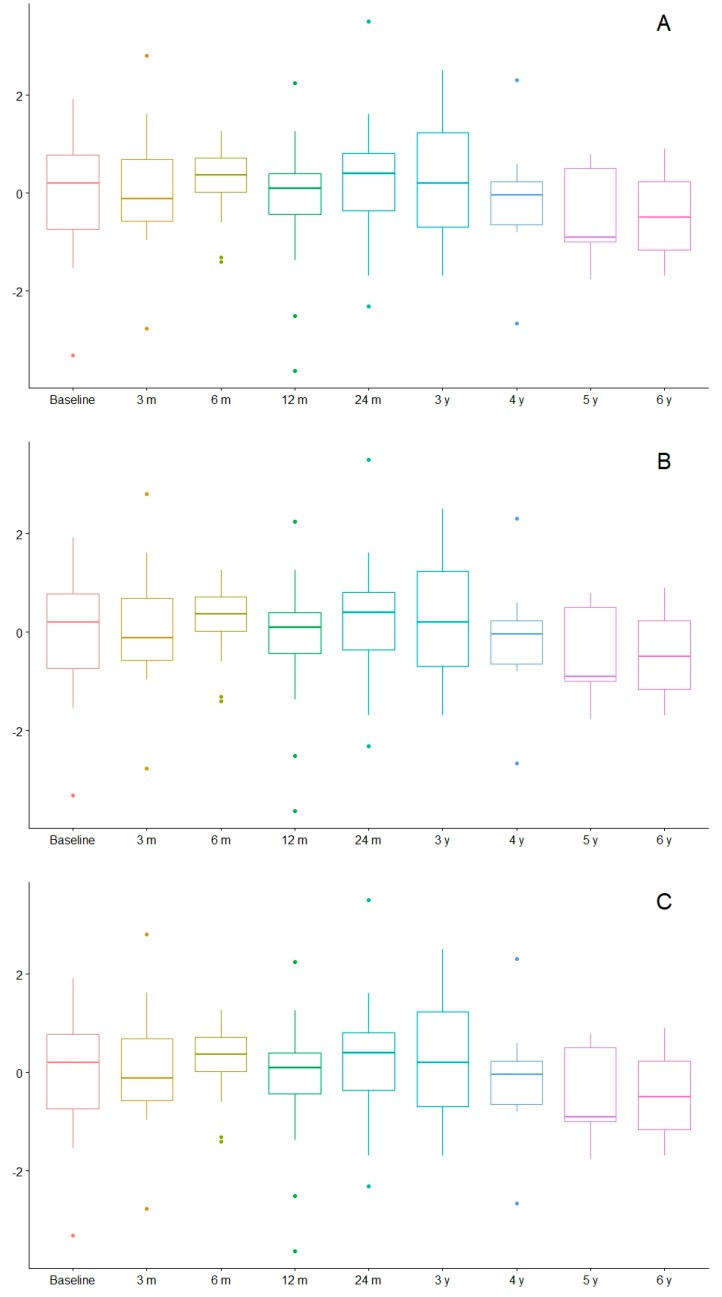
Evolution of weight (**A**), height (**B**), and BMI (**C**) in z-score values. m: months, y: years.

In Figure 2, the children and their different ranges of BMI are represented throughout time. Most were found to be adequately nourished in every check-up. Amongst the children older than 5, there was one child who was mildly malnourished at 3, 12, and 24 months, and one case of severe malnutrition at 12 months. There was one overweight patient at 3 months and at 3 years, and 2 cases at 6 and at 24 months. Obesity criteria was achieved by two children at 3 years on the diet, and one child at 4 years on the diet. Amongst the group younger than 5 years old, there was one case of mild malnutrition after 4 years on the diet, two cases affected by overweight at 3 and at 12 months on the diet, and one with obesity at 24 months. The evolution of the z-score of weight, height, and BMI throughout time is shown in Figure 3.

The low levels of prealbumin and RBP were 3 at 3 months (2 mild and 1 moderate), 5 at 6 months (all mild), 12 at 12 months (10 mild and 2 moderate), 13 at 2 years (9 mild, 4 moderate), 5 at 3 years (3 mild, 1 moderate, 1 severe), 6 at 4 years (4 mild and 2 moderate), 3 at 5 years (2 mild and 1 moderate), and 3 at 6 years (1 mild, 1 moderate, 1 severe). In just one case was the level of prealbumin lower than 10 mg/dl, whereas the rest of the moderate and severe deficiencies corresponded to the RBP levels. 

No patient presented either vitamin B12 or a folic acid deficiency. The lowest levels of ferritin were detected in an isolated case at month 3 (4 ng/mL), at year 2 (3 ng/mL), and at year 3 (6 ng/mL). Even though no actual deficiencies were observed, there were several abnormalities in the blood count of several patients throughout the follow-ups. The mean corpuscular volume augmented over 90 fl in one patient at 3 months; four at 6 months; five in the first and second year; three at 3 years; and one at 4, 5, and 6 years. The neutrophils were lower than 1500/mcl in two cases at 3 and at 6 months, three cases at 12 months, and one at 2 and at 3 years. One case of eosinophilia was described at month 3 and at month 6, and two at month 12, and again just one at years 2 and 3. Although no other causes were found for these abnormalities, there was no binding reason to acknowledge that the cause could be, in fact, the KD. 

Vitamin E stayed within normal ranges during the entire follow-up, six patients had vitamin A deficiency (1 at 12 months (0.18 mg/L), one at 2 years (0.19), two at 3 years (0.16 and 0.19), and two at 4 years (0.17 and 0.12)), and six had vitamin D deficiency (one at 3 months (19.3 ng/mL), two at 12 months (16.0 and 4.9), two at 2 years (12.7 and 13.5), and one at 4 years (19.0). None had calcium or PTH alterations. No patient had a magnesium deficiency. 

Low levels of carnitine were observed in two cases (at month 6 with levels of total carnitine at 20 microM/L, and at month 12 with levels of L-carnitine at 7.6 microM/L). Two children registered low levels of zinc (one at 12 months (64 mcg/dL) and one at 4 years (54.8)), and three had selenium deficiency (one at 6 months (53 mcg/L), one at year 2 (68.4), one at year 4 (59.2)). 

**Figure 4:**
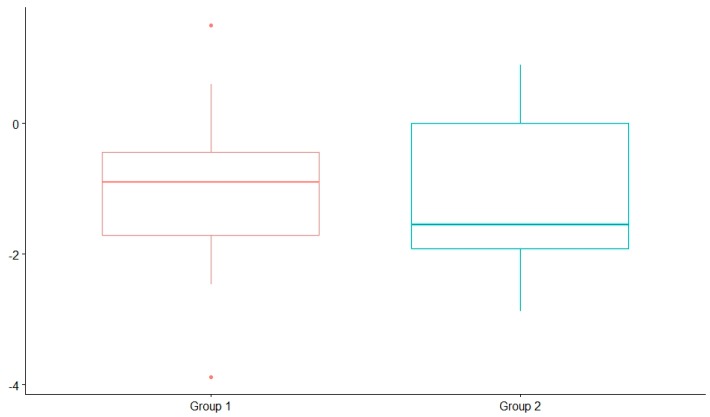
z-score eight comparison after 2 years on the diet; group 1: children older than 2 years, group 2: children younger than 2 years.

The average and standard deviation of the weight, height, and BMI z-score are presented in Table 5. The height z-score was significantly lower after 2 years on the diet compared with before the diet. However, there were no significant differences in the weight and BMI z-score. No significant differences were found in the weight, height, and BMI z-score after 2 years on the diet among those children who started before 2 years of age and those who started after 2 years of age. Nevertheless, height was mildly influenced after 2 years on the diet in the children who started before the age of 2 years (Figure 4). The coefficients of variation were 13.7% (in those children who started a KD after the age of 2 years) and 8.9% (in those who started before). 

## 5. Discussion

Multiple studies have confirmed the efficacy of the KD. In our cohort, approximately half of the patients with drug-resistant epilepsy became seizure-free and about 60%–75% of the patients achieved a decrease of over 90%. Our results show more success than the data published from other hospitals because our study only included information on children who followed a KD for a prolonged period. Those patients that respond well to the treatment were precisely those who maintained the diet longer. Those in which the diet was not completely efficient abandoned the diet early on.

Because of the accumulated past data on certain syndromes and clinical cases where the KD is shown to be more efficient and beneficial, it is recommended that the diet be implemented as soon as possible. Some of these clinical disorders are Doose syndrome [15,16] and Otahara syndrome [42,43], GLUT-1 deficiency [20], PDH [21], and alterations of complex-1 of the respiratory mitochondrial chain [44]. In this study there were eight cases in which GLUT-1 deficiency was either suspected or received the actual diagnosis, one case of Doose syndrome, and one case of Otahara syndrome. All of them had a 100% recovery rate. The three cases with mitochondrial diseases in this study had a positive response above 90%. Infants as a group also responded favorably to the KD. There was a 90% improvement in the four children who started on the KD before 12 months of age.

The average level of blood ketone bodies was adequate over the follow-up. However, some patients presented low levels of ketone bodies that were not related to a worse diet response, and thus the KD is probably effective due to other mechanisms besides ketosis.

Side effects in these children were mostly mild and did not require the termination of the diet. The digestive issues they presented have been described in about 50% of patients on a KD [22,45]. In this study, the digestive symptoms were less noted, whereas the most frequent was constipation, which was successfully managed with polyethylene glycol in all cases in which the bowel regimen and diet were insufficient. Amongst the urinary symptoms, hypercalciuria was observed in almost one fourth of the children. There was only one case in which the child suffered nephrocalcinosis (3.8%), which corresponded to the same frequency shown in literature that indicates 3%–7% [46,47,48]. The singular case was diagnosed 12 months after the KD was implemented and was probably associated to a mutation in the sodium channels. All cases of hypercalciuria were treated with potassium citrate (1–2 mEq/kg/day divided in three doses), which reduces the lithiasis risk in patients on a KD, and normalizes or improves calciuria [49].

The lipid profile underwent frequent changes, especially during the first 12 months of the diet. These variations have been described in up to 60% of children [22], and may happen during the first month [50] but tend to normalize within the first few months following the diet’s introduction [24,51]. There has been no increased cardiovascular risk associated with the diet in recent studies [52]. There have been cases of liver dysfunction in children on the KD, especially in association with valproic acid treatment [44,53]. Amongst our patients, seven children presented mild liver enzyme conditions and five of them received valproic acid treatment.

KD treatment for more than 2 years has been associated with an increased risk of bone fracture and development issues [24]. One patient suffered a bone fracture while practicing sport after 7 years on the KD, (a bone densitometry after 6 years on the KD was on file for the child with a z-score of −1.096).

Several studies have shown that children treated with KD for more than 6 months showed a decreased growth rate [29,30,31,54,55,56], especially in younger children, although the decrease they present is reversed after the patients are taken off the KD [57]. In our global cohort, we also observed a gradual reduction of the height z-score over time, especially after 12 months of KD. In children who began the diet before 2 years of age, a more acute decline in height development was observed. Potential causes of poor growth in children on the KD include inadequate intakes of energy and protein (compared with recommended values) the effects of the underlying diseases and treatments, ambulatory status, acidosis/ketosis, and related endocrine changes [32,33]. More research is required to identify children at higher risk of growth delay. Results showed weight was also affected, although differences were not statistically significant. The BMI stayed stable throughout the diet due to the paralleled decline in growth in both height and weight.

KD produces modifications in the levels of micronutrients. Vitamin A and E, for the most part, are not affected, however the following are: vitamin D [58], magnesium, selenium [59,60] (sometimes associated to a cardiomyopathy [61]), and carnitine. The latter is especially vulnerable when on the KD for a prolonged period, when the child is very young, or when valproic acid is used as part of the treatment [25,26]. In our cohort, the most frequent micronutrient deficiencies before KD were vitamin D and selenium. After the diet was implemented, there were six cases of mild deficiency of vitamins A and D. Only two children had carnitine deficiency—both were younger than 4 years and neither were being treated with valproic acid. It is our opinion that no more carnitine deficiencies were found in children in treatment with valproic acid because they were already being supplemented before the KD was implemented. Two children presented zinc deficiency and three children presented selenium deficiency, but none had myocardium dysfunction symptoms. When a deficit was detected, supplements were prescribed according to RDA, increasing the doses if necessary, to normalize plasma levels. No patients registered low magnesium levels. No patients suffered anemia, even though there were several cases of corpuscular volume growth, eosinophilia, and neutropenia.

In children who achieved higher than 50% reduction of seizures on the KD, they were weaned off the KD after approximately 2 years. However, in children where the almost complete disappearance of seizures is achieved and only mild side effects are suffered, the KD diet can be maintained many years, with no time limit, weighing, of course the benefits and the risks [62]. In some pathologies it is the treatment of choice and must be maintained for a prolonged period. In GLUT-1 deficiency, for example, the KD is recommended at a minimum until puberty, and until adulthood in many cases because of its effectiveness [63,64]. In our study, all patients followed a KD for more than 2 years because of their excellent response to the diet. It should be noted that the most frequent diagnosis from our cohort (7/26) was GLUT-1 deficiency.

The benefits of a CKD can be appreciated in the long run, even after the patient has been weaned off the diet [24,65]. Guidelines suggest that, in patients in which the CKD is effective and necessary in the long run, should, after several years, change to a less restrictive diet such as the MAD or LGID [62]. In our study, 11 patients started out with a DAM, 4 patients who had a CKD switched to a MAD, and 4 patients went from a MAD to a LGID.

## 6. Conclusions

In conclusion, the KD is an efficient and safe treatment during childhood. It is especially efficient in certain pathologies. Generally, the nutritional status of the patients on KD is good, even when they are on the diet for an extended period. The nutritional deficiencies are mild and easy to treat with nutritional supplements. Height is usually affected in prolonged treatments; however, it is difficult to determine if the KD is exclusively responsible given the patient’s pathology, the severity of the condition, and the need for other treatments. Side effects in the long term are mild and easy to treat. In children on a KD treatment for an extended period, it is possible to transition to less restrictive diets (MAD, LGID) that allow for a better treatment adherence.

## Figures and Tables

**Table 1:** Characteristics of the patients in the study.

	Patients with KD (*n* = 26)
Sex	17 M (65.38%)
	9 F (34.62%)
Type of seizure	GTC, 1 (4%)
	Myoclonic, 7 (28%)
	Tonic, 4 (16%)
	Focal onset, 5 (20%)
	Atonic, 1 (4%)
	Typical absences, 1 (4%)
	Clonic, 1 (4%)
	Various, 5 (20%)
Median age of epilepsy debut (range)	0.97 years (2 days to 4 years)
Median age at introduction of KD (range)	4 years (3 months to 12.6 years)
Type of KD	CKD 3:1, 11 (42.31%)
	CKD 4:1, 4 (15.38%)
	MAD, 11 (42.31%)
Administration route	Oral, 21 (80.77%)
	Nasogastric tube, 2 (7.69%)
	Gastrostomy (G-tube), 3 (11.54%)
Median length of KD (range)	3.91 years (2–9.4 years)
Present situation	Off of the diet, 10 (38.5%)
	Active, 16 (61.5%)

KD: ketogenic diet, M: male, F: female, GTC: generalized tonic-clonic, MAD: modified Atkins diet, CKD: classic ketogenic diet.

**Table 2:** Etiology of epilepsy.

Etiology	Frequency	Percent	Specific Etiology
Unknown	4	16.00	
Metabolic	10	40.00	7 GLUT1DS
			3 mitochondrial diseases
Structural	5	20.00	1 cortical dysplasia
			4 hypoxic-ischemic structural brain abnormalities (2 perinatal problems, 1 meningitis, 1 sepsis)
Genetic disorders	4	16.00	3 gene disorders:
			1 KCNQ2 gene mutation
			1 CDKL5 gene mutation
			1 Noonan syndrome (PTPN)
			1 chromosome disorders:
			1 Down syndrome
Infection	2	8.00	1 herpes simplex virus
			1 Streptococcus pneumoniae

GLUT1DS: glucose transporter type 1 deficiency syndrome, *KCNQ2*: potassium voltage-gated channel subfamily Q member 2, *CDKL5*: cyclin dependent kinase-like 5, *PTPN*: protein tyrosine phosphatase non-receptor.

**Table 3:** Effectiveness of the diet according to the etiology of epilepsy. The number of patients within each group that were within each improvement category is represented according to the reduction of epileptic seizures compared to baseline.

	1 Year			2 Years			3 Years		
	50%–90%	90%–100%	100%	50%–90%	90%–100%	100%	50%–90%	90%–100%	100%
Unknown	-	-	3/4	-	-	3/4	-	1/2	1/2
Metabolic	1/10	1/10	7/10	-	1/10	9/10	-	1/7	6/7
Structural	1/5	1/5	2/5	2/5	-	2/5	-	1/1	-
Genetic disorders	-	1/4	3/4	-	1/4	3/4	-	-	2/2
Infection	1/2	1/2	-	2/2	-	-	2/2	-	-

**Table 4:** Number of patients who suffer from late side effects of the KD at every evaluation period.

Time on the Diet	3 Months	6 Months	12 Months	2 Years	3 Years	4 Years	5 Years	6 Years
Total of patients suffering side effects	15/26	13/26	21/26	22/26	11/18	7/12	3/8	3/6
Constipation	4	3	3	2	1	-	2	1
Abdominal Pain	-	-	-	1	-	-	-	-
Nausea/vomiting	1	1	1	1	-	1	-	-
Diarrhea	-	-	-	1	-	-	-	-
Hypoglycemia	-	-	-	-	-	1	-	-
Hypercholesterolemia	6	5	10	8	6	4	1	1
Hypertriglyceridemia	3	1	2	2	2	-	-	1
Elevated AST, ALT or GGT	3	2	1	2	1	-	-	-
Hypercalciuria	6	6	9	11	4	3	-	1
Protein in urine	1	4	3	3	1	1	1	-
Fatigue	1	-	-	-	-	-	-	-
Anorexia	1	-	-	-	-	-	-	-
Hyperuricemia	1	-	2	4	3	2	1	1
Metabolic acidosis (pH)	2	-	4	4	2	-	-	-

AST: aspartate aminotransferase, ALT: alanine aminotransferase, GGT: gamma glutamyl transpeptidase.

**Table 5:** Variations of weight, height, and BMI of the z-score.

	Basal	2 Years on KD	Paired Differences	
	Median ± SD		Median ± SD	*p*-Value *
	(Minimum–Maximum)			
Weight	−0.252 ± 1.10	−0.477 ± 1.33	−0.257 ± 1.297841	0.3869
	(−2.65 – 1.95)	(−3.31 – 3.00)		
Height	−0.279 ± 1.21	−1.03 ± 1.27	−0.644 ± 1.431242	0.04681
	(−2.4 – 2.14)	(−3.88 – 1.5)		
BMI	−0.00364 ± 1.21	0.197 ± 1.25	0.248 ± 1.630604	0.5046
	(−3.31 – 1.91)	(−2.31 – 3.5)		

* With statistical significance, value of *p* < 0.05. BMI: body mass index, KD: ketogenic diet, SD: standard deviation.
